# Bilateral Cranial Cruciate Ligament Rupture Treatment in a Dog Affected by Classical Ehlers–Danlos Syndrome

**DOI:** 10.3390/vetsci12121162

**Published:** 2025-12-05

**Authors:** Núria Vizcaíno-Revés, Stefan J. Rietmann, Elisabet Domínguez, Dolors Fondevila, Vidhya Jagannathan, Tosso Leeb, Mar Bardagí

**Affiliations:** 1 Hospital Clínic Veterinari, Universitat Autònoma de Barcelona, 08193 Bellaterra, Spain; nuria.vizcaino@uab.cat (N.V.-R.); elisabet.dominguez@uab.cat (E.D.); 2 Institute of Genetics, Vetsuisse Faculty, University of Bern, 3001 Bern, Switzerland; stefan@rietmann-online.de (S.J.R.); vidhya.jagannathan@unibe.ch (V.J.); 3 Graduate School for Cellular and Biomedical Sciences, University of Bern, 3001 Bern, Switzerland; 4 Animal Medicine and Surgery Department, Universitat Autònoma de Barcelona, 08193 Bellaterra, Spain; dolors.fondevila@uab.cat (D.F.); derma.veterinaria@gmail.com (M.B.); 5 Anicura Ars Veterinària Hospital Veterinari, 08034 Barcelona, Spain

**Keywords:** *Canis lupus familiaris*, surgery, orthopedics, dermatology, genetics, collagen, skin, whole genome sequencing, hereditary, inherited

## Abstract

**Simple Summary:**

We report an 11-month-old Maltese dog that was diagnosed with cranial cruciate rupture. The dog also had Ehlers–Danlos syndrome, a hereditary disease that causes weakness of connective tissues. Genetic analysis of the dog revealed a new heterozygous missense variant in the *COL5A1* gene encoding the collagen type V alpha 1 chain. An increased risk for cranial cruciate ligament rupture has been reported in human patients with Ehlers–Danlos Syndrome but not in dogs. We provide the first report of such an event in dogs and subsequent stifle stabilization.

**Abstract:**

Ehlers–Danlos syndrome (EDS) is a congenital disorder affecting connective tissue. Patients diagnosed with EDS may present with joint instability, and in human medicine, an increased risk of cranial cruciate rupture has been described. A few therapeutic options for these patients have been described, with no evident superiority of one technique due to small study groups. Cranial cruciate rupture has never been described in a dog with EDS. This is a case report of an 11-month-old Maltese diagnosed with bilateral cruciate ligament rupture and classical EDS with a previously undescribed heterozygous *COL5A1* missense variant that underwent stifle stabilization.

## 1. Introduction

Ehlers–Danlos syndrome (EDS) is a heterogeneous group of inherited multiorgan disorders that affect the connective tissue, leading to a variety of dermatological, musculoskeletal, and cardiovascular manifestations. It is characterized by joint hypermobility or instability, skin hyperextensibility, easy bruising, wound healing abnormalities, and tissue fragility [1,2]. Currently there are 13 clinical types of EDS recognized in human medicine and an additional EDS-like type. Most types have a genetic etiology with pathogenic variants in genes that encode fibrillar collagens of types I, III, and V. Genes that have a role in the synthesis of glycosaminoglycan might also be involved [3].

In human medicine, knee disorders are a common manifestation in EDS. While these primarily involve patellar instability, an increased risk of anterior cruciate ligament injury and meniscal tears has also been described [2,4].

Joint laxity and dislocation are common in humans affected by EDS; however, they are less frequently found in dogs [5,6,7,8,9,10,11]. Patella luxation and coxofemoral luxation have been reported in dogs with EDS [10], but a cranial cruciate rupture has not yet been reported in dogs with EDS.

The surgical treatment of knee instability in human patients with EDS is considered a challenge. The biomechanical differences in the collagen-containing tissues of these patients remain largely unknown. A few different techniques have been described in isolated cases, but large population studies are lacking; therefore current evidence is not strong enough to establish any clear treatment recommendations [12,13].

To the best of the authors’ knowledge, we provide the first report of a successful surgical treatment of a dog with a confirmed diagnosis of EDS with joint and skin hyperextensibility and bilateral cruciate ligament rupture.

## 2. Case

An 11-month-old male Maltese dog was presented to the neurology department for the evaluation of right hind limb lameness. Clinical signs had started approximately 5 weeks prior, and there was no history of associated trauma. The lameness had initially been treated with prednisone by the referring veterinarian, and the owners reported an improvement following the treatment. Four days before presentation, the patient showed reluctance to exercise and, on the day of presentation, a recurrence of the right hind limb lameness.

Upon presentation its vital signs were within normal limits. The patient showed a non-weight-bearing lameness of the right hind limb. The neurological examination revealed pain in sciatic nerve palpation, bilaterally. The orthopedic examination revealed bilateral stifle joint effusion and a positive drawer test. The rest of the neurological, orthopedic, and clinical exams were unremarkable.

Stifle radiographs, as well as lumbosacral magnetic resonance imaging, were recommended, and in the meantime, the patient was started on meloxicam 0.1 mg/kg once daily per os and gabapentin 10 mg/kg every 8 h per os.

A pre-anesthetic CBC and chemistry panel revealed lymphocytosis at 6.64 K/µL (1.05–5.10), mild eosinophilia at 1.52 K/µL (0.06–1.23) and basophilia at 0.5 K/µL (0–0.1).

**Figure 1:**
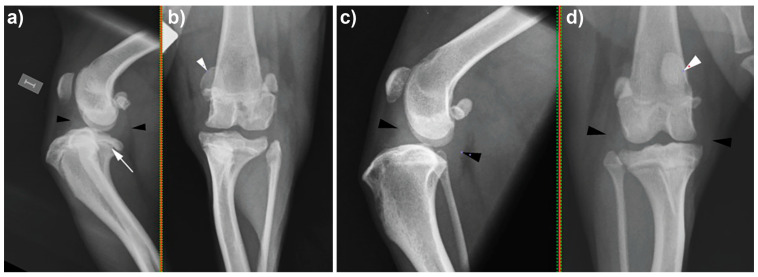
Stifle radiographs. (**a**) Mediolateral and (**b**) caudocranial radiographs of the left stifle. There is caudodistal sloping of the femorotibial joint and remodeling of the tibial plateau (long white arrow), as well as cranial displacement of the tibia relative to the femur. Increased soft tissue opacity in the joint space (black arrowheads) causing narrowing of the infrapatellar fat pad and displacement of the fascial planes is visible. The patella is medially displaced (white arrowhead). Periarticular new bone formation in the patella, femoral trochlea, medial and lateral epicondyles, and on the lateral and medial surfaces of the tibial condyles can be seen. (**c**) Mediolateral and (**d**) caudocranial radiographs of the right stifle. Black arrowheads show increased soft tissue opacity within the joint space, and the white arrowhead points to the medial displacement of the patella.

Radiographs of both stifle joints (Figure 1) revealed caudodistal sloping of the femorotibial joint space—worse on the left side—and remodeling of the tibial plateau. The tibiae were cranially displaced relative to the femurs; there was increased soft tissue opacity in the joint space, causing narrowing of the infrapatellar fat bodies and displacement of the fat opaque fascial planes. The patellae were medially displaced, and periarticular new bone formation was detected bilaterally, presenting worse on the left side. The combination of radiographic findings was compatible with tibial plateau deformans, joint effusion, and osteoarthrosis suggestive of cranial cruciate/meniscal disease, as well as medial patellar luxation. The changes were bilateral but more severe in the left pelvic limb.

High-field MRI of the lumbosacral vertebral column (Canon Vantage Elan 1.5T scanner, Canon Medical Systems Corporation, Otawara-shi, Tochigi, Japan) was performed. The protocol included pre- and postcontrast sequences, following intravenous administration of a dose of 0.1 mmol/kg gadoteric acid (Clariscan 0.5 mmol/mL, GE Healthcare Bio-Sciences, S.A.U. Madrid, Spain). Dorsal short-tau inversion recovery (STIR) and T2-weighted (T2w) sequences, sagittal T2w and T1w post-contrast sequences, and transverse T2w, T2* gradient recalled echo, T1w, and T1W post-contrast sequences revealed no structural abnormalities in the vertebral column.

The lameness was therefore considered of orthopedic origin, and surgical stabilization of both stifles was recommended. Staged bilateral extracapsular stabilization was recommended and performed 8 weeks apart. Briefly, after a lateral approach to the stifle, an arthrotomy was performed. Rupture of the cranial cruciate ligament was visually confirmed. The ruptured fibers of the ligament were debrided, and both menisci were inspected. The stifle joint was stabilized by means of an extracapsular suture (Fiberwire, Arthrex, Munich, Germany) as previously described [13]. Thereafter, the joint was flushed, and the site was closed by layers. Both surgeries were reported uneventful.

During the first hospitalization, two cutaneous wounds of unknown origin were detected, and during the second hospitalization, three new cutaneous wounds were found. The patient recovered well from both surgeries, showing an improvement in lameness over the next few weeks.

**Figure 2:**
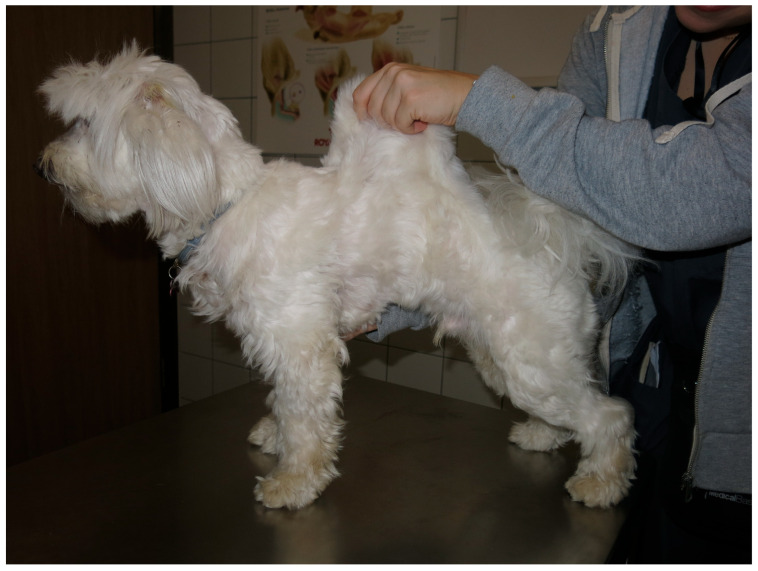
Hyperelastic skin phenotype. The photo shows manual extension of a fold of dorsolumbar skin of the patient to measure the skin extensibility index.

Two months after the second stifle surgery, the patient was presented to the dermatology department. The guardians reported that the patient’s skin was very fragile and that it ruptured easily when the animal was combed. Dermatological examination revealed hyperextensible skin (Figure 2). Healed skin from previous wounds did not look atrophic. The extensibility index [vertical height of dorsolumbar skin fold/body length) × 100] was 26% (Figure 2).

**Figure 3:**
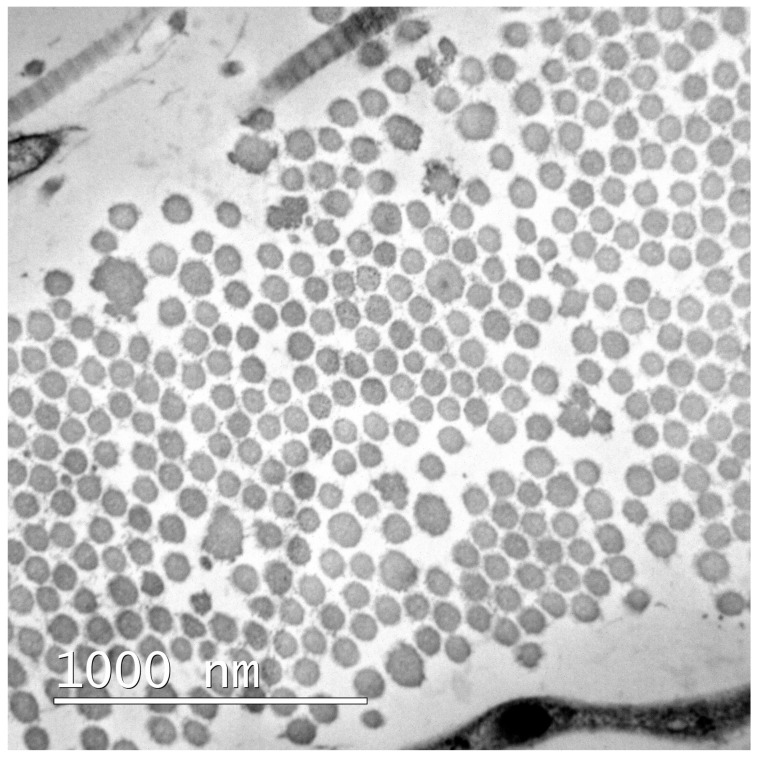
Transmission electron microscopy of a skin biopsy. Cross-section through a collagen fiber in which there is a marked variation in the collagen fibrils, irregular and serrated collagen fibrils’ outlines, and irregular interfibrillar spaces.

The dog presented carpal and tarsal hyperextension, as well as carpal valgus which had not been present in the previous examinations. The rest of the exam, including ocular and oral examinations, did not reveal any abnormality. Skin biopsies were performed, but no microscopic lesions were detected either on hematoxylin/eosin or Masson trichrome stains. Skin samples were taken afterwards to perform transmission electron microscopy (TEM). Cross-sections of collagen fibers showed a marked variation in the diameter of the collagen fibrils, abnormal and serrated collagen fibril outlines, and irregular interfibrillar spaces (Figure 3). Taking both the joint and skin hyperextensibility with a skin extensibility index above 14.5% and TEM findings, EDS was diagnosed. An echocardiographic study was performed, in which morphology, kinetic flow, and transvalvular flow were within normal limits, and no large vessel dilation was observed.

For the genetic investigation, an EDTA blood sample was taken from the affected dog, and genomic DNA was isolated to prepare a PCR-free library, which was subsequently sequenced with 2 × 150 bp reads on an Illumina instrument at 22× coverage. The resulting paired-end sequencing data was aligned to the UU_Cfam_GSD_1.0 genome reference assembly, and variant calling was performed as previously described [14]. The sequencing data is publicly available at the European Nucleotide Archive under the accession number SAMEA118215569. For private variant filtering, the sequencing data from the case were compared to 1664 control genomes, resulting in 43 heterozygous variants and no homozygous variants private to the EDS-affected dog with a predicted protein-changing effect (Tables S1 and S2). Visual inspection of these variants identified a heterozygous missense variant in the *COL5A1* gene that encodes the collagen type V alpha 1 chain and is a well-known candidate gene for classical EDS (cEDS), both in humans and animals alike [15,16]. The variant NC_049230.1:g.50,854,306G>A or XM_038548863.1:c.2228G>A is predicted to result in the exchange of a glycine residue to a glutamate residue in the encoded protein, XP_038404791.1:p.(Gly743Glu), at a position that is highly conserved throughout evolution. This exchange affects a Gly-X-Y repeat motif, in which the glycine residues at every third position are essential to enable trimerization and formation of the triple helical structure of the mature collagen fibril. An exchange of glycine in the Gly-X-Y repat motifs is therefore likely to interfere with collagen multimer formation and secretion of the protein from the cell, typically leading to a dominant negative effect [17]. There are several reports of dogs with EDS presumably based on the same pathomechanism [18,19]. The genetic findings allowed us to refine the diagnosis to classical EDS (cEDS).

The guardians of the dog were advised on an appropriate lifestyle to prevent trauma and to seek veterinary attention in case of non-healing wounds or any other skin diseases. Five years after bilateral stifle stabilization and the diagnosis of EDS, both joints remain stable, and the patient shows no lameness. The guardians only report sporadic episodes in which the patient sits down and is reluctant to walk. Regarding the skin, the guardians have managed most of the skin wounds that have appeared, which have been commonly small and have rapidly healed, some of them with atrophic scars. The skin of the peribucal skin and dorsal head has become thinner and more atrophic than it was during the first consultation.

## 3. Discussion

This is the first case report describing the treatment of bilateral cruciate rupture in a dog with cEDS. Extracapsular stabilization resulted in good limb function and long-term stifle joint stability.

Cranial cruciate ligament rupture is considered the most common cause of lameness in dogs [20]. Several reports have tried to identify risk factors of cranial cruciate ligament rupture in veterinary medicine. Spay/neuter status and body weight have been identified as risk factors [21]. Bilateral cruciate ligament rupture has been found to be more frequent in males, young dogs (mean age of 4 years), overweight patients, and Rottweilers [22].

EDS has been identified as a risk factor for cranial cruciate rupture in human medicine [2,4]. However, such a risk has not been described in veterinary patients, and to the best of the authors’ knowledge, no cases have been published so far.

In human patients, major and minor clinical criteria along with molecular testing to identify the causative variant(s) in the respective gene(s) and/or TEM findings are needed to diagnose EDS [1,15]. In dogs, no clinical criteria have been published, and despite the different pathogenic variants that have been detected [18,19,23], the diagnosis of the ED-like syndrome so far mostly relies on clinical findings, a skin extensibility index above 14.5%, and light microscopic findings. However, because collagen may appear normal on light microscopy, full documentation can require ultrastructural and biochemical studies [24]. The present case fulfilled the clinical, the skin extensibility index, and ultrastructural findings to diagnose the ED-like syndrome. Furthermore, we identified a previously undescribed heterozygous *COL5A1* missense variant that confirmed and refined the previous diagnosis to cEDS. Such variants are commonly due to de novo mutation events [1], and we speculate that this is the case here as well. However, as no samples from the parents were available, we could not experimentally confirm the presumed de novo mutation event. As whole-genome sequencing becomes increasingly more accessible, genetic analysis presents a non-invasive alternative to more traditional diagnostic approaches and should be considered, especially in diseases with phenotypic variability and clearly established candidate genes such as EDS (with the exemption of hypermobile EDS) [1]. In fact, in human patients, the definitive diagnosis of most subtypes of EDS relies on molecular confirmation with identification of the causative variant(s) in the respective gene [1]. In the canine patient reported here, the molecular diagnosis of cEDS matched with the clinical criteria of cEDS in humans, fulfilling the major clinical criteria (skin hyperextensibility, atrophic scarring, and joint hypermobility) and two minor criteria (skin fragility and the cruciate ligament rupture as a suspected complication of joint hypermobility) [1]. Other complications described less commonly in humans with cEDS, like ocular, gastrointestinal, or cardiovascular signs [15], have not been detected in our patient during the 3-year follow up.

EDS in humans has been associated with a few skeletal abnormalities, such as genu recurvatum [25]. The canine patient in this case report had a bilateral premature closure of the proximal tibial growth plate, which is also known as the tibial plateau. This has been associated with a higher predisposition to cranial cruciate ligament rupture [26]. Whether the tibial deformity, EDS, or the combination of both predisposed the patient to bilateral cruciate ligament rupture remains unknown. Due to the late diagnosis of EDS, biopsies of the cruciate ligament were not sampled during the surgery, and further intervention to obtain samples was considered unethical.

In human medicine, there are few reports on successful outcomes of patients with EDS and anterior cruciate ligament deficiency. The reported treatment options include synthetic ligament reconstruction, autograft hamstring reconstructions, and physeal sparing iliotibial bands with allografts [12,27,28,29]. Studies involving large numbers of patients are lacking, and therefore there are no conclusive studies contemplating the best treatment choice for cruciate ligament deficiency. However, the treatment of joint instability is considered potentially challenging because of the reduced biomechanical characteristics of the collagen-containing anatomical structures.

Whether a corrective osteotomy would have been a more appropriate technique for stifle stabilization in this patient remains unknown. Studies on long-term results of extracapsular repair (ERC) compared to corrective osteotomies such as tibial tuberosity advancement or a tibial plateau leveling osteotomy (TPLO) have concluded that TPLO but not ERC patients achieve normal function when compared to a normal group, and therefore a TPLO is considered a more appropriate recommendation for most patients [30]. Nevertheless, the surgical risk must be accounted for. In human medicine, controversy exists regarding the bone quality and bone healing potential of patients with EDS. While fractures are not part of the diagnostic criteria of the disease, lower bone mineral density and bone quality (measured by trabecular bone score) in adults affected by the disease are detected. Furthermore, an increase in prevalence of vertebral fractures has been detected among affected individuals [31,32]. It is unknown if veterinary patients show similar bone changes, if these might vary with age, and if they could have an impact on the surgical risk of a corrective osteotomy.

Although an increased intraoperative complication rate has been reported in human patients with EDS [33], no complications appeared in the dog reported herein. Meticulous hemostasis and gentle soft tissue retraction are key to avoiding complications such as wound dehiscence, wound infection, or hematoma development [33].

While both stifles were considered stable on palpation after surgery, the guardians reported that the patient sporadically showed unwillingness to walk. Pain is a common feature of EDS in humans, which may be attributed to hypermobility and dislocation or might be neuropathic in origin [34,35]. A postoperative multimodal pain management plan is considered a prominent aspect of treatment. Since the episodes of reluctancy to walk were sporadic in our patient, the guardians decided against chronic medical treatment. Advanced gait analysis of the patient was not performed because it was not available at our institution.

EDS was diagnosed after the stifle stabilization surgeries had been completed in this patient. Long delays in diagnosis or underdiagnosed patients are also a big concern in human medicine, where patient, disease, provider, and system factors are considered to play a role in this delay [33,36]. Better knowledge of the disease, a multidisciplinary approach, and future improvements in diagnosis might help with the early detection of EDS-affected patients.

## Data Availability

The original contributions presented in this study are included in the article/Supplementary Material. Further inquiries can be directed to the corresponding author.

## Supplementary Materials

The following supporting information can be downloaded at https://www.mdpi.com/article/10.3390/vetsci12121162/s1, Table S1. Accession numbers of whole genome sequences from 1665 dogs; Table S2. Private variants in the sequenced dog with Ehlers–Danlos syndrome.
